# Cryptosporidiosis Associated with Wildlife Center, Scotland

**DOI:** 10.3201/eid1605.091468

**Published:** 2010-05

**Authors:** Christopher C. McGuigan, Kirsty Steven, Kevin G.J. Pollock

**Affiliations:** National Health Service Tayside, Dundee, Scotland, UK (C.C. McGuigan); Perth and Kinross Council, Perth, Scotland, UK (K. Steven); Health Protection Scotland, Glasgow, Scotland, UK (K.G.J. Pollock)

**Keywords:** enteric infections, outbreak, cryptosporidiosis, parasites, protozoa, wildlife park, zoonoses, letter

**To the Editor:** Handwashing is the single most important prevention step in reducing transmission of gastrointestinal zoonoses (*1*). Nevertheless, Health Protection Scotland receives reports of 500 to 700 laboratory-confirmed cases of cryptosporidiosis each year. Cryptosporidiosis symptoms include profuse, watery diarrhea, often accompanied by bloating, abdominal pain, and nausea. On April 15, 2005, NHS Tayside District’s public health department called a meeting of the incident control team after a single index case of cryptosporidiosis in Scotland. One reported case rarely results in such measures; however, initial investigations determined that this case-patient may have acquired infection by contact with scouring (diarrhea) lambs at a wildlife center, during the Easter break (March 27–April 10, 2005). Subsequent public health actions included active surveillance of recent *Cryptosporidium* spp. laboratory reports, active case finding, the microbiologic analysis of feces/rectal swabs from lambs and bedding samples, and an assessment of the wildlife center’s private water supply. Control measures included the removal of lambs from the center, disinfection of the premises with hypochlorite, and stopping direct contact between animals and visitors.

In total, 128 microbiologically confirmed cases were reported to the incident control team. An additional 252 clinical cases were reported among wildlife center visitors for whom no stool sample was taken. The illnesses of these persons had a similar implied incubation period (typically 6–7 days) and their age profiles were the same as patients with laboratory-confirmed cases. Of 128 patients with confirmed cases, 117 visited the wildlife center, and infections of the remainder were attributed to secondary spread. Most case-patients were Tayside residents and were generally resident in towns and villages near the wildlife center. Of the 128 human isolates, 103 were identified as *Cryptosporidium parvum*. Oocysts from the environmental samples (lamb pen drain and central drain debris) were also identified as *C. parvum*. Isolates could not be obtained from lambs because the lambs had died and were subsequently incinerated by the wildlife center. Although assessment of the private water supply revealed unacceptable levels of coliforms, oocysts were not detected.

Daily gate receipts for the wildlife center were obtained. Using these as a denominator for confirmed cases, we calculated the daily attack rate. The attack rate peaked at 8.1% on April 8, 2005. The relative risk for visiting the wildlife center over the defined period was estimated to be ≈13.3 for confirmed *Cryptosporidium* infection. In view of the strength and clarity of the association between visiting the wildlife center (petting lambs in particular) and being a case-patient (Figure), no formal analytical epidemiologic investigation was conducted.

**Figure:**
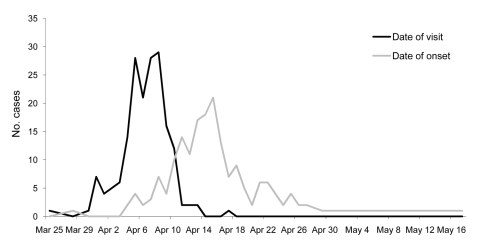
Date of onset of cryptosporidiosis cases reported to Health Protection Scotland and date of visit to wildlife center, 2005.

These results suggest that the outbreak was caused by direct contact with scouring lambs, a recognized risk factor for cryptosporidiosis, coupled with inadequate handwashing facilities (*2*,*3*). Anecdotal reports indicate that children were encouraged to pick up lambs from the farm enclosure, despite visible signs of diarrhea on the animals. The lack of handwashing facilities in this wildlife park was surprising because the Scottish government had conducted an information campaign that Spring (March), encouraging primary prevention initiatives, specifically in petting farms and zoos, and recommending the provision and use of handwashing facilities (www.infoscotland.com/handsclean/CCC_FirstPage.jsp). Moreover, no handwashing facilities were located near the lamb-petting area, and considerable effort was required to locate a handwashing basin in the wildlife center complex. Several alcohol hand sanitizers were located on site, but the microbicidal effects on *Cryptosporidium* spp. are insufficient to prevent infection, especially after direct contact with livestock (*4*,*5*).

After publication of the outbreak report, an assessment of handwashing and hygiene facilities elsewhere in Scotland found them to be suboptimal and that stronger education, regulation, and other control measures were needed to protect the public. Recent *Escherichia coli* O157 outbreaks in England have accentuated the unresolved issues for UK petting farms concerning hand hygiene and zoonotic infections (*6*).
